# Lead (Pb) Exposure Enhances Expression of Factors Associated with Inflammation

**DOI:** 10.3390/ijms19061813

**Published:** 2018-06-20

**Authors:** Emilia Metryka, Karina Chibowska, Izabela Gutowska, Anna Falkowska, Patrycja Kupnicka, Katarzyna Barczak, Dariusz Chlubek, Irena Baranowska-Bosiacka

**Affiliations:** 1 Department of Biochemistry and Medical Chemistry, Pomeranian Medical University, Powstańców Wlkp. 72, 70-111 Szczecin, Poland; emilia_metryka@o2.pl (E.M.); zara1988@wp.pl (K.C.); anula05@gmail.com (A.F.); patrycjakupnicka@o2.pl (P.K.); dchlubek@sci.pam.szczecin.pl (D.C.); 2 Department of Biochemistry and Human Nutrition, Pomeranian Medical University, Broniewskiego 24, 71-460 Szczecin, Poland; izagut@poczta.onet.pl; 3 Department of Conservative Dentistry and Endodontics, Pomeranian Medical University, Powstańców Wlkp. 72, 70-111 Szczecin, Poland; katarzyna.barczak@pum.edu.pl

**Keywords:** lead (Pb), inflammatory processes, cytokines, interleukins

## Abstract

The human immune system is constantly exposed to xenobiotics and pathogens from the environment. Although the mechanisms underlying their influence have already been at least partially recognized, the effects of some factors, such as lead (Pb), still need to be clarified. The results of many studies indicate that Pb has a negative effect on the immune system, and in our review, we summarize the most recent evidence that Pb can promote inflammatory response. We also discuss possible molecular and biochemical mechanisms of its proinflammatory action, including the influence of Pb on cytokine metabolism (interleukins IL-2, IL-4, IL-8, IL-1b, IL-6), interferon gamma (IFNγ), and tumor necrosis factor alpha (TNF-α); the activity and expression of enzymes involved in the inflammatory process (cyclooxygenases); and the effect on selected acute phase proteins: C-reactive protein (CRP), haptoglobin, and ceruloplasmin. We also discuss the influence of Pb on the immune system cells (T and B lymphocytes, macrophages, Langerhans cells) and the secretion of IgA, IgE, IgG, histamine, and endothelin.

## 1. Introduction

The current abundance and toxicity of lead (Pb) makes it the second most dangerous environmental poison, according to the Agency for Toxic Substances and Disease Registry’s Priority Substance List [1]. Although leaded petrol has been removed from use in many countries (e.g., in 1976–1986 in the United States of America (USA), and in 2005 in the European Union (EU), Pb compounds are still being used in aviation fuels. In addition, although the sale of lead paints was banned in the USA in the late 1970s, and in the EU in 1992, they are still used in the restoration and preservation of historic buildings and art [2,3,4]. Importantly, no lowest safe concentration exists for Pb, which contributes to 0.6% of the global burden of disease [5].

Pb has been shown to exert a negative effect on the immune system—a key element in inflammation, consisting of defensive reactions to injury in a living organism [6]. The basic elements of the cellular immune response to an inflammatory factor are monocytes/macrophages, B and T lymphocytes, natural killer (NK) cells, and granulocytes [7]. Inflammatory reactions also crucially depend on humoral factors, which include plasma coagulation; kinin and complement systems; intracellular chemicals (histamine, serotonin, and lysosomal enzymes); and specific inflammatory mediators, such as prostaglandins, leukotrienes, platelet activating factor (PAF), and cytokines (TNF-α, IL-1, IL-2, IL-6, IL-8, IL-15, IFNγ). The mediators responsible for the inflammatory process can be subdivided into early mediators (TNF-α, IL-1, IL-6, IL-8), known as activators, and secondary mediators (arachidonic acid metabolites, proteases, reactive oxygen species—ROS, nitric oxide—NO), known as effector mediators, which are directly responsible for functional and structural damage to cells. Also crucial for the development of the inflammatory process is the endothelium of blood vessels, whose activation by inflammatory mediators is well documented [8,9].

The immune system seems to be one of the more sensitive targets of Pb. Although at environmental low concentrations, Pb is not able to cause overt damage to the main immune cells, and does not result in the deficiencies of immune cells that are determined by routine tests, it does adversely affect the regulation and function of immune cells [10]. In our previous review, we presented recent literature on the effect of Pb on inflammation in the brain, particularly the expression of selected cytokines, activity of enzymes, and expression of receptors participating in inflammatory processes [11]. We also presented evidence that exposure to Pb may result in microgliosis and astrogliosis by triggering a signaling cascade and the production of proinflammatory cytokines [11]. In the present review, we summarize the most recent evidence that Pb can cause generalized inflammation in the body, and discuss possible molecular and biochemical mechanisms of its proinflammatory action.

## 2. The Effect of Pb on Cytokines

Cytokines are bioactive proteins produced by many cells of the immune system. They are key mediators of the inflammatory response; influencing the interaction and communication between cells; and stimulating their movement towards the sites of inflammation, infections, and injuries. 

### 2.1. Interleukins 2 and 4 (IL-2 and IL-4)

Interleukin-2 (IL-2) is a cytokine indispensable for proper growth, proliferation, and differentiation of T lymphocytes. It directs the development of ThO lymphocytes towards Th1 [12]. Interleukin-4 (IL-4), on the other hand, acts extensively on B lymphocytes and is one of the most important factors stimulating the production of IgE-class antibodies. It induces T-cell proliferation and enhances Th2 differentiation [13].

The effect of Pb on the levels of IL-2 and IL-4 in mouse blood serum was investigated by Iavicoli et al. (2006) [14]. For this purpose, female Swiss mice were given a feed containing lead acetate (PbAc) at different concentrations. Administration began the first day after copulation, which was considered the first day of pregnancy of the mice. The same feed that was given during the pregnancy was continued during lactation, and was also given to the offspring for nine months following weaning. At the end of the exposure, blood levels of lead (Pb-B) in the offspring and possible changes in serum IL-2 and IL-4 were measured. Significant increases in IL-4 production were observed at higher concentrations of Pb in the diet (40 and 400 ppm), although with a significant decrease in IL-2 production. Importantly, the lowest concentration of Pb in the diet (0.02 ppm), which corresponded to a level of Pb-B of 0.8 μg/dL, increased production of IL-2 and significantly decreased IL-4 production [14]. According to the authors of the study, these results provide evidence of a reversal of cytokine production depending on blood Pb levels [14].

### 2.2. Interleukin 8 (IL-8)

Interleukin (IL-8) is a proinflammatory cytokine and a potent chemotactic agent for neutrophils that is produced by many types of cells, including epithelial and endothelial cells [15]. In numerous in vitro and in vivo models, IL-8 has been implicated in angiogenesis and metastasis [16].

In a study by Lin et al. (2015), human gastric adenocarcinoma cells (AGS cells) were stimulated with 0.1 μM Pb(NO_3_)_2_, which induced activation of the *IL-8* gene [17]. A previous study by the same team of researchers showed that the promoter region of gene *CXCL8* (IL-8 protein is encoded by the *CXCL8* gene) comprises transcription factors, such as activator protein 1 (AP-1), nuclear factor kappa B (NF-κB), and nuclear factor for IL-6 expression (NF-IL6) [18]. Therefore, authors decided to analyze these transcription factors [17,19] and demonstrated that transcription factor AP-1 was involved in the activation of the Pb-induced *IL-8* gene, whereas the NF-κB transcription factor played only a minor role. In addition, Pb at a concentration of 0.1 μM in AGS cells also induced over-expression of the c-jun protein, a component of heterodimeric protein transcription factor AP-1 [17].

It has been previously suggested that the epidermal growth factor receptor (EGFR) associated with p42/44 MAPK (mitogen activated protein kinase) plays an important role in Pb-induced inflammatory reactions [20,21]. Lin et al. (2015) aimed to determine whether EGFR also plays a role in the activation of the Pb-induced *IL-8* gene, and exposed AGS cells to EGFR inhibitors (PD153035 and AG1478) [17]. The use of these EGFR inhibitors resulted in a decrease in *IL-8* gene activation, but EGFR inhibitors were unable to fully abolish Pb-induced *IL-8* gene activation, which means that other receptors or channels may also play an important role in these signal transduction pathways [17]. The level of p42/44 MAPK phosphorylation induced by Pb was also reduced by both EGFR inhibitors, which indicates that EGFR is involved in the Pb-induced expression of the *IL-8* gene, and p42/44 MAPK may mediate this signal transduction pathway [17].

In order to confirm the contribution of p42/44 MAP kinase to the Pb-induced activation of the *IL-8* gene, Lin et al. (2015) conducted further studies in which 10 μM of selective inhibitor of MAP kinase kinase (MEK)—PD98059—was administered to AGS cells. MEK is also a p42/44 MAPK activator, and earlier studies had shown that p42/44 MAP is a kinase that is often observed to be involved in *IL-8* gene activation [17,18]. The use of the MEK inhibitor (PD98059) resulted in a significant inhibition of *IL-8* gene activation, which confirms the role of p42/44 MAP kinase in the Pb-induced activation of the *IL-8* gene because MEK is a p42/44 MAPK activator, and its inhibition—as described above—results in a significant blocking of *IL-8* gene expression.

**Figure 1:**
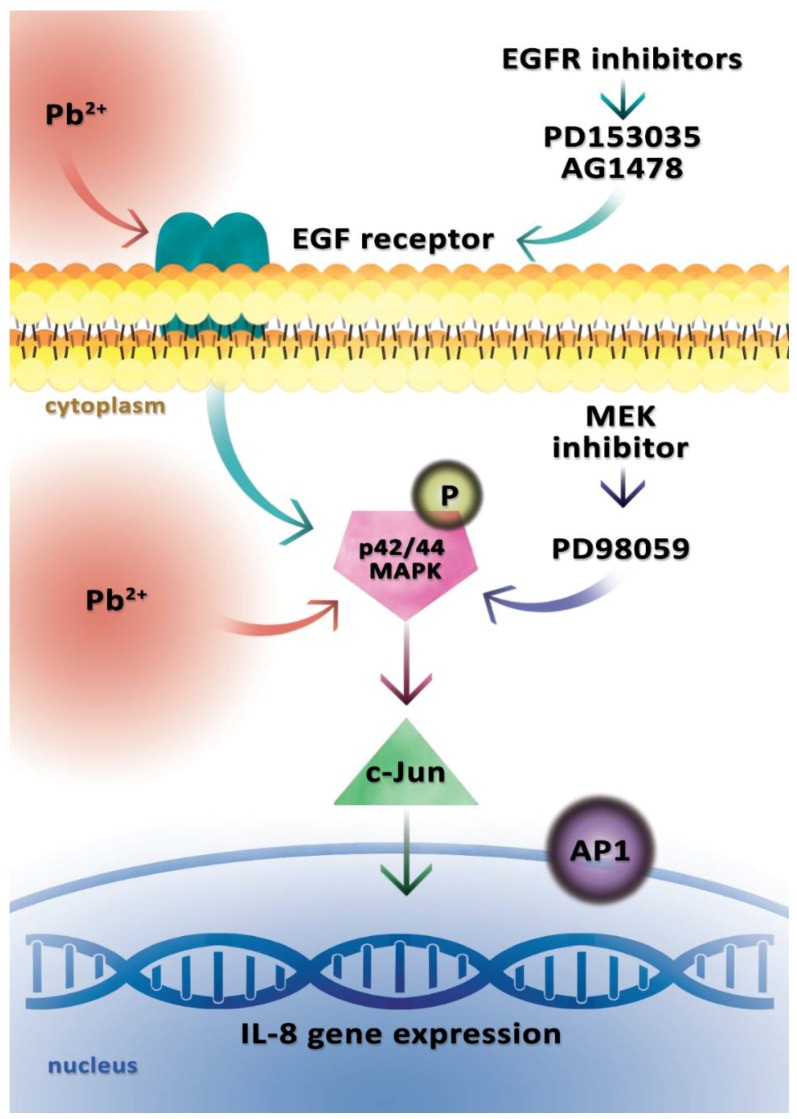
Model of lead (Pb^2+^)-induced activation of the *IL-8* gene. The diagram presents a signaling pathway leading to the induction of the *IL-8* gene in human AGS cells (human caucasian gastric adenocarcinoma) after the administration of 0.1 μM Pb(NO_3_)_2_. Pb^2+^ activates the epidermal growth factor receptor (EGFR) and p42/44 mitogen activated protein (MAP) kinase phosphorylation, which then activates the protein heterodimeric transcriptional factor AP-1 (containing the c-jun protein), resulting in the activation of *IL-8* gene expression. The use of EGFR inhibitors (PD153035 and AG1478) resulted in a decrease in *IL-8* gene expression, but EGFR inhibitors were unable to fully abolish Pb(NO_3_)_2_-induced *IL-8* gene expression. The use of a MAP kinase kinase (MEK) inhibitor (PD98059) significantly suppressed *IL-8* gene expression.

In a study by Lin et al. (2015), gastric cancer cells reacted to as little as 0.1 μM Pb(NO_3_)_2_ [17]. Endothelial cells produced IL-8 in response to 0.5 μM of Pb(NO_3_)_2_, whereas peripheral blood mononuclear cells stimulated with PbAc exhibited *IL-8* gene activation at Pb concentrations above 10 μM [17,22,23]. Those observations suggest that the Pb dose required for IL-8 induction depends on the type of cell, and that chronic exposure to even low Pb doses may result in local IL-8 production and inflammation in the gastrointestinal tract, and in adverse conditions in predisposed individuals, it may contribute to the development of gastric cancer (Figure 1) [17].

Another study was conducted to explain the mechanisms underlying the increased synthesis and secretion of IL-8 in endothelial cells isolated from a human umbilical vein in response to Pb at a concentration of 50 μmol/L. It indicated the influence of Pb on transcriptional factor Nrf2 [23]. Inactive Nrf2 is bound to the Kelch-like ECH-associated protein 1 (Keap1) and is found in the cytoplasm [23]. Activation of Nrf2 (e.g., by heavy metal ions) results in a dissociation from Keap1 and migration to the cell nucleus, where it forms a heterodimer with a small Maf protein and binds to the antioxidant response element (ARE) of nuclear DNA via the leucine zipper [24].

Nrf2 is responsible for the induction of xenobiotic-metabolizing enzymes (XMEs), including NQO1 (NAD(P)H/quinone oxidoreductase) [23]. Zeller et al. (2010) observed the up-regulation of NQO1 in endothelial cells in response to Pb at 50 μmol/L, indicating the activation of Nrf2 signaling pathway [23].

**Figure 2:**
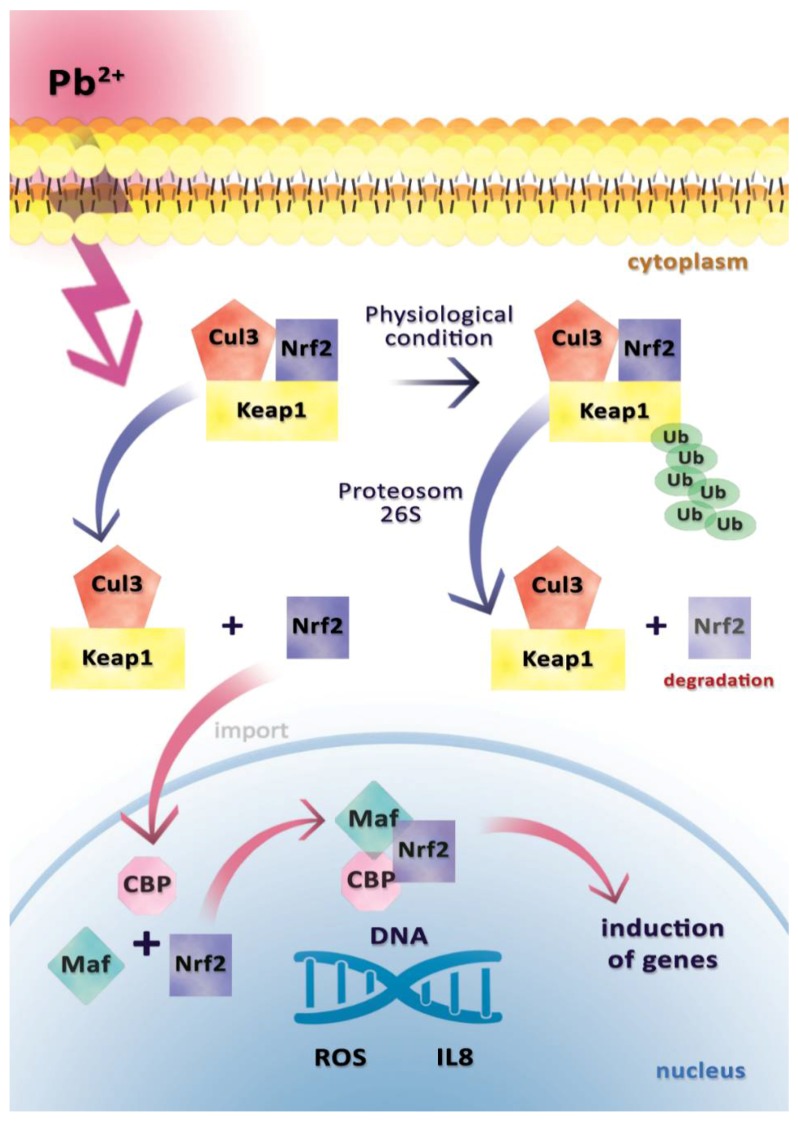
Mechanism of activation and inactivation Nrf 2 by Pb^2+^. In physiological conditions, Nrf2 binds to its inhibitor, Kelch-like ECH-associated protein 1 (Keap1). This is followed by the dimerization and then sequestration of Nrf2 in the cytoplasm. The Keap1–Nrf2 complex connects ubiquitin (via the Cul 3-dependent ligase) and is recognized by the 26S proteosome, where the Nrf2 transcription factor is degraded. After exposure to Pb^2+^, Nrf2 is released from the Keap1–Nrf2 complex, and translocated to the nucleus, where a small Maf protein attaches forming a heterodimer and CREB-binding protein (CBP). The incorporation of the newly formed complex into DNA initiates the transcription of antioxidant response genes and *IL-8* gene results in enhanced reactive oxygen species (ROS) and IL-8 synthesis.

Another study showed that the promoter region of the *IL-8* gene contains an antioxidant response element (ARE) that is bound to Nrf2, as mentioned earlier [25]. Therefore, to confirm the key role of Nrf2 in the Pb-induced IL-8 production in endothelial cells, Nrf2 knockdown was performed through siRNA administration. It completely blocked the transcription, translation, and secretion of Pb-induced IL-8. Finally, Zeller et al. (2010) demonstrated that Pb stimulates IL-8 synthesis and secretion in a mechanism dependent on Nrf2 (Figure 2) [23].

The relationship between exposure to Pb and increased IL-8 concentration has also been presented by Yang et al. (2014), who examined Pb levels in the blood of children living near a Pb refinery (Longchang, a city in Sichuan, China) [26]. The study involved 88 unrelated healthy children, divided according to authors of this paper into those with elevated and low blood Pb, that is, according to the U.S. Centers for Disease Control and Prevention (CDC) threshold [27] (44 children with Pb ≥ 10 μg/dL and 44 children with Pb < 10 μg/dL, although the most recent CDC recommendations suggest that the permissible threshold level for children is Pb < 5 μg/dL, CDC 2012) [28]. A higher level of IL-8 was shown in the group of children with elevated blood Pb levels [26].

### 2.3. Interleukins 1b and 6 (IL-1b and IL-6)

Interleukins 1b and 6 are potent proinflammatory cytokines inducing prostaglandin synthesis, neutrophil influx, and activation and proliferation of T and B lymphocytes. Both are mainly synthesized by monocytes and macrophages. They stimulate the synthesis of acute phase proteins and are responsible for increasing body temperature during infection [29].

Dyatlov and Lawrence (2002) investigated how exposure to Pb intensified the symptoms of a bacterial infection in BALB/c mice receiving Pb acetate at 0.5 mM from birth [30]. After 21 days, the juveniles were separated from their mothers and were still given the same Pb acetate solution in drinking water. On day 22, the mice were infected with *Listeria monocytogenes*, and blood serum IL-1b and IL-6 concentrations were measured. Pb significantly increased the infection-induced increase in serum IL-1b when compared with the infection in mice not exposed to Pb. The exposed mice also had a significant increase in serum IL-6 levels [30]. In order to confirm that Pb was responsible for the severity of the symptoms, artificial conditions imitating the infection were created by administering IL-1b and/or IL-6 directly to the mice. The severity of symptoms was observed only in mice injected simultaneously with IL-1b and IL-6. The experiment showed that Pb exacerbated the occurrence of mouse listeriosis symptoms. As the authors point out, the results suggest that children with elevated levels of Pb in the blood may show prolonged and more intense symptoms of bacterial infections [30].

In another study, the team evaluated the effect of Pb on the expression of cytokine genes. The research was carried out in the brain tissue of murine newborns whose mothers received a 0.1 mM solution of lead acetate from the beginning of pregnancy until the 21st day after giving birth. The concentration was selected based on previous studies, showing that concentration taken during pregnancy and lactation resulted in blood Pb levels of 15–20 μg/dL [31], which corresponds to the amount of Pb in the blood of children exposed to environmental pollution [32].

Cytokine expression was tested at 21 days after birth using microarrays, Real-time reverse transcription PCR (real-time RT-PCR), Luminex, and enzyme-linked immunosorbent assay (ELISA). A significant effect of Pb on the expression of TGF-β1 and IL-6 was found in the whole studied brain tissue. The expression of IL-6 mRNA increased in both males and females. That effect increased in the group of females when the concentration of lead acetate increased to 0.5 mM [33]. The increased expression of *TGF-β1* and *IL-6* genes, which persisted during the strong development of the body, may have an adverse effect on the growth and differentiation of neurons [34]. 

Kishikawa et al. (1998) had previously shown that Pb exposure increases mRNA and IL-6 protein levels in the brains of adult mice treated with lipopolysaccharide (LPS) [35].

### 2.4. Interferon Gamma (IFNγ)

IFNγ is an important cytokine for innate and adaptive immunity to viral and some bacterial and protozoal infections. It is an important activator of macrophages and an inducer of the expression of molecules of the major histocompatibility complex (MHC) class II [36].

A study by Yücesoy et al. (1997) unequivocally confirmed that occupational exposure to lead can result in a significant decrease in IFNγ [37]. In addition, in many other studies of exposure to Pb resulted in decreased concentrations of IFNγ in plasma [38], serum [39], and splenocytes of rodents [40], and in Th1 cell clones [38,41], but the subcellular mechanism underlying the inhibition of IFNγ production by Pb has not been investigated.

As cellular immunity is largely dependent on IFNγ, it is important to understand the mechanism by which the Pb-induced inhibition of IFNγ production interferes with cellular immunity [42]. For this purpose, Heo et al. (2007) conducted a study to clarify the effect of Pb on IFNγ mRNA expression, kinetics of IFNγ protein biosynthesis, IFNγ protein secretion, and proteasome degradation [42]. They demonstrated that PbCl_2_ (at 25 μM) did not affect mRNA expression in Th1 cell clones, which suggests that the suppression of IFNγ production by Th1 cells when exposed to PbCl_2_ is not due to a decreased transcription of IFNγ [42]. In the same experiment, IFNγ production was significantly reduced upon addition of Pb (at a concentration of 25 μM) both in Th1 cell supernatants and cell lysates when compared with the control, clearly ruling out the inhibition of secretion as a cause of the Pb-induced suppression of IFNγ production [42]. Increased intracellular degradation of IFNγ was not responsible for the lower levels of IFNγ, as the administration of lactacystin to Th1 cells (at concentrations of 10, 100 nM, and 1 µM), which is the most selective inhibitor of proteasome proteolysis, did not prevent the loss of IFNγ [42]. However, the study revealed that exposure of Th1 cells to Pb, resulting in reduced synthesis of IFNγ, may be due to selective inhibition in the initial translation stages (where protein production is not inhibited by Pb) [42]. 

In another study by Kamińska et al. (1998), the ingestion of 5 mg Pb/kg body weight once a day for four weeks was used to estimate the effect of Pb on IFNγ levels in cows [43]. Blood was collected prior to the initial treatment with Pb and again after 3, 7, 14, 21, and 28 days for the determination of plasma IFNγ levels [43]. The effect of Pb on IFNγ levels in the cows depended on whether it was determined in plasma or leukocytes. IFNγ increased in plasma but dropped in ex vivo leukocytes, which shows that Pb can exert a dysregulatory effect on IFNγ levels in the blood [43]. 

Radbin et al. (2014) investigated the influence of Pb and Cu on the expression of IFNγ and interleukin 4 (IL-4) [44], and for this purpose, adult BALB/c mice were given a solution of Cu(NO_3_)_2_ at 2 mg/L or Pb(NO_3_)_2_ at 0.3 mg/L. After 15 days of the experiment, spleen samples were collected from the mice to determine the expression of *IL-4* and *IFNγ* genes. The authors of the study showed an increased expression of the *IFNγ* gene in both groups. However, that increase was more significant in the Pb group. *IL-4* gene expression increased in the group of animals receiving Cu, whereas in Pb-treated mice, the expression of this cytokine decreased [44].

### 2.5. Tumor Necrosis Factor Alpha (TNF-α)

TNF-α is a multifunctional cytokine that exerts pleiotropic biological effects in various tissues. It is produced mainly by activated macrophages and lymphocytes at the site of inflammation, and together with IL-6 and IL-1, it participates in local and systemic inflammatory reactions [29].

In a study by Guo et al. (1996), human peripheral blood mononuclear cells (PBMCs) collected from healthy donors were treated with 1 ng/mL of lipopolysaccharide (LPS) in the presence of various concentrations of PbCl_2_ (0, 10, and 50 μM) in order to assess the impact of Pb on the expression of TNF-α and soluble forms of tumor necrosis factor receptors (TNF-Rs), and to understand the mechanisms responsible for the increased expression, uptake, and reactivity of TNF-α [45]. 

**Figure 3:**
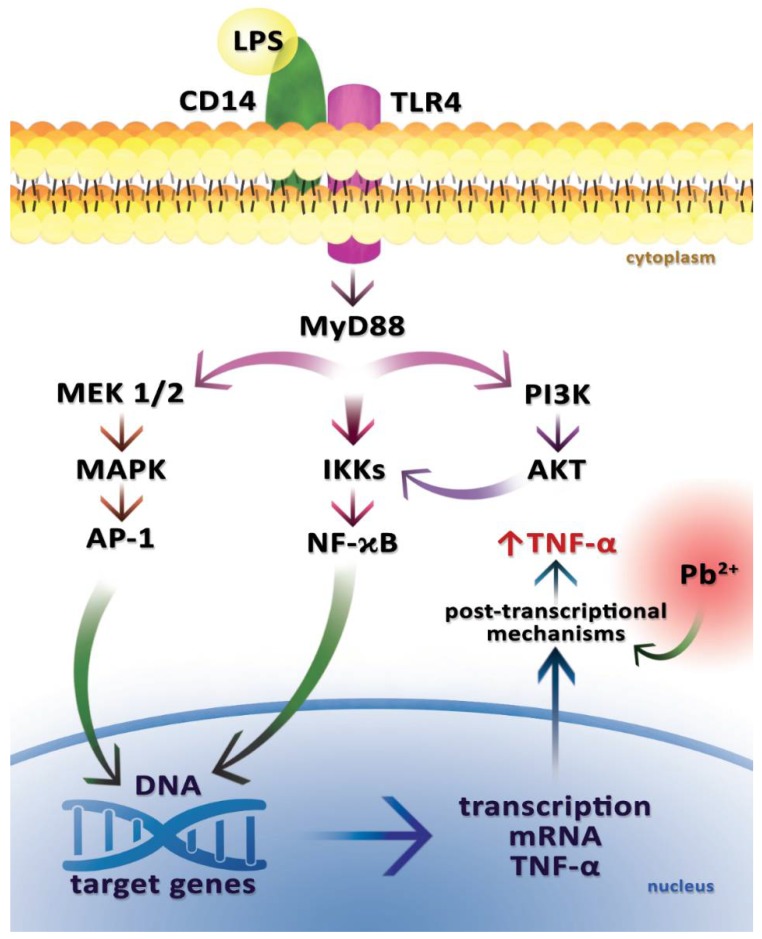
The signaling pathway that results in the production of tumor necrosis factor alpha (TNF-α) in macrophages after the stimulation by lead and lipopolysaccharide (LPS). Lead increases the expression of TNF-α in LPS-treated cells via post-transcriptional mechanisms. It thus increases the effect of lipopolysaccharide (LPS) that is uptaken by the CD14 receptor (cluster of differentiation 14) and its co-receptor TLR 4 (Toll-like receptor TLR 4). The diagram shows some of the transmitters potentially mediating the regulation of TNF-α expression. The signal is transmitted via the MyD88 adapter protein (myeloid differentiation primary response gene 88). One of the activated pathways is the MAPK pathway. MEK1/2 (mitogen-activated protein kinase kinase), MAPK (mitogen-activated protein kinases), and activator protein 1 (AP-1) are activated. The NF-κB factor (nuclear factor kappa B) activation pathway is also stimulated by the regulation of IKKs (IκB kinase) activity. The resulting AP-1 and NF-κB lead to enhanced transcription of TNF-α mRNA. PI3K–AKT pathway is also activated. Attachment of LPS to the receptor leads to the phosphorylation of PI3K (phosphoinositide 3-kinase), which then activates AKT (protein kinase B). There is a mutual adjustment between the NF-κB and PI3K–AKT signaling pathways. The activation of the above-mentioned signaling pathways by LPS and Pb results in enhanced TNF-α synthesis.

In that experiment, Pb caused an increase in total TNF-α cell expression in PBMC (+1 ng/mL LPS), but it did not affect the level of TNF-α mRNA, both in the presence and absence of 1 ng/mL of LPS, indicating that increased TNF-α expression occurs via post-transcriptional mechanisms (Figure 3) [45].

The third prime untranslated region (3′-UTR) of TNF-α mRNA contains the AUUUA sequence, which normally represses translation, but this repression may be abolished by phosphorylation of the protein that binds the AUUUA sequence elements via mitogen-activated protein kinase (MAPK)–CSBP (cytokinin specific binding protein) kinase pathway, which results in the release of the binding protein and thereby suppresses the inhibitory effect on TNF-α translation [9]. Pb has been shown to activate protein kinase C in rat brains, and so it may also have an effect on CSBP kinase activation [46]. In addition, in a study by Guo et al. (1996), Pb increased the expression of TNF-α in PBMC (+LPS) through post-transcriptional mechanisms [45].

There are two forms of TNF-α: soluble sTNF-α and membrane-bound mTNF-α. A study by Guo et al. (1996) showed that Pb can also increase the secretion of TNF-α protein by PBMC (+LPS) by increasing the conversion of factor-α to sTNF-α [45]. The soluble form is responsible for a significant part of the biological activity of TNF-α, including stimulation of the inflammatory response. That increased conversion may be due to a Pb-induced increase in the activity of the metalloproteinase converting enzyme or an increase in TNF-α protein synthesis [45]. TNF-α works via two types of tumor necrosis factor receptor: TNF-R1 (CD120α, p55, p60) and TNF-R2 (CD120β, p75, p80) [45]. TNF-R1 is responsible for cytotoxicity and many detrimental aspects of TNF-α, while TNF-R2 can facilitate TNF-α binding to TNF-R1 [47]. Both types of this receptor can be decomposed by TACE (tumor necrosis factor alpha converting enzyme) (metalloproteinase) to form so-called soluble forms (sTNF-R1, sTNF-R-2) that are natural TNF-α inhibitors [48]. In an experiment carried out by Guo et al. (1996), Pb increased TNF-α uptake and reactivity by increasing TNF-R p55 expression, but it did not show any effect on TNF-R p75 expression [45]. Ultimately, the experiment showed that Pb affected the expression of TNF-α and TNF-R in human peripheral blood mononuclear cells (PBMCs). All of the aforementioned mechanisms may be responsible for the severity of organ damage and mortality in experimental animals exposed to LPS and Pb, and administration of the anti-TNF-α antibody can fully protect experimental animals against both LPS- and Pb-dependent mortality [45].

Abundant evidence indicates that heavy metals may affect the activity of LPS, a potent inducer of TNF-α in vivo and in vitro [49,50]. Animal studies have shown that Pb increases mortality caused by LPS [51]. The current hypothesis for LPS-induced mortality is the massive production and release of TNF-α by many different cells, including macrophages, monocytes, and T and B lymphocytes. For example, 50% of human macrophages undergoing in vitro stimulation of LPS showed surface TNF-α expression after 24 h [52]. LPS binding by CD14 receptor and TLR4 receptor triggers multiple signaling pathways that activate NF-κB and p42/44 mitogen-activated protein kinase (MAPK), leading to the expression of proinflammatory cytokines, including TNF-α.

Cheng et al. (2006) conducted a study to identify the cells responsible for the overproduction of TNF-α following co-exposure to LPS + Pb, and to determine the role of protein kinase C (PKC) and p42/44 MAPK in the induction of this production [50]. Liver damage in mice was treated as a biological endpoint to investigate the mechanism of increased TNF-α induced by LPS + Pb. Hewett et al. (1993) also found that high doses of LPS resulted in excessive production of TNF-α and consequently liver damage [53]. TNF-α induces the apoptosis of hepatocytes by Fas-associated proteins with death domain (FADD) signaling, resulting in caspase activation (the blocking of TNF-α production or TNF-α signaling pathways with caspase inhibitors reduces TNF-α-induced hepatic injury) [54,55,56]. 

In the study by Cheng et al. (2006), A/J mice were intraperitoneally administered with Pb (100 μmol/kg), LPS (5 mg/kg), Pb + LPS (Pb 100 μmol/kg, LPS 5 mg/kg), or saline control. To determine serum TNF-α levels, blood was taken 1.5 h after intraperitoneal administration and again 24 h later to measure the levels of aspartate transaminase (AST) and alanine transaminase (ALT), the markers of liver damage. Serum TNF-α levels were undetectable in the A/J mice receiving saline or only Pb. The LPS-treated mice had a small amount of TNF-α in serum (<250 pg/mL) and showed a slight increase in AST (300 U/L) and ALT (30 U/L). The mice receiving Pb + LPS had serum TNF-α levels of about 2 ng/mL, which confirms the significant effect of Pb on LPS-induced TNF-α production. The mean levels of AST and ALT were also significantly elevated in the Pb + LPS group, at 720 U/L and 600 U/L, respectively, indicating severe liver damage [50]. As Comalada et al. (2003) demonstrated that LPS induces TNF-α-induced apoptosis in macrophages too [57], Cheng et al. (2006) wanted to confirm the relationship between TNF-α and hepatic injury through intraperitoneal injection of pentoxifylline (PTX) to the A/J mice (PTX being a potent inhibitor of TNF-α transcription in vivo) [50]. The mice that were given PTX at 100 mg/kg (1 h before Pb + LPS administration) showed lower blood TNF-α levels when compared with those not given PTX. AST and ALT levels were also significantly reduced after exposure to PTX.

Monocyte/macrophages are the main sources of TNF-α in inflammatory and infectious conditions [58]. In order to confirm whether macrophages/monocytes are involved in liver damage caused by co-exposure to Pb + LPS, Cheng et al. (2006) inactivated macrophages/monocytes with GdCl_3_ (40 mg/kg) 24 h prior to Pb + LPS administration [50]. GdCl_3_ caused a decrease in serum TNF-α levels in mice that received Pb + LPS. It also reduced serum AST and ALT levels. It demonstrated that monocytes/macrophages are responsible for the production of excess TNF-α in co-exposure to Pb + LPS, which causes liver damage. The inactivation of these follicular cells and blocking of signaling pathways for TNFα production can be effective in alleviating liver damage [50].

Cheng et al. (2006) also demonstrated that p42/44 MAPK phosphorylation increased in peritoneal macrophages and RAW264.7 cells (murine macrophage cell line) in the Pb + LPS group, indicating that p42/44 MAPK signaling pathways are involved in increasing TNFα expression caused by co-exposure to Pb + LPS [50]. To confirm the contribution of PKC and p42/44 MAPK in the induction of TNF-α expression, mice were intraperitoneally given an MEK1 and MEK2 inhibitor (U0126 25 μmol/kg) and a PKC inhibitor chelerythrine chloride (C21H18NO4Cl 5 mg/kg) before exposure to Pb, LPS, or Pb + LPS. Both U0126 and C21H18NO4Cl significantly suppressed TNF-α expression induced by Pb + LPS and effectively decreased serum AST and ALT levels. Thus, the use of the PKC and p42/44 MAPK inhibitors showed that PKC and p42/44 MAPK are involved in Pb + LPS-induced TNF-α expression in peritoneal macrophages and RAW264.7 cells. 

In addition, Cheng et al. (2006) also demonstrated that Pb (10 μM) significantly increased TNF-α expression induced by low LPS doses (0.1 to 1 ng/mL) in murine peritoneal macrophages isolated by rinsing the peritoneal cavity of A/J mice in 10 mL of sterile phosphate buffer. Pb also increased the expression of TNF-α in RAW264.7 cells after administration of LPS at 1 or 10 ng/mL, with the effect being more pronounced for 10 μM Pb than for 1 μM Pb [50]. 

To sum up, Cheng et al. (2006) showed the synergistic effect of Pb + LPS (co-exposure of mice to Pb + LPS strongly induced TNF-α expression in macrophages in the whole blood and peritoneal macrophages) [50]. Although separate administration of Pb or LPS only slightly increased MAPK phosphorylation in peritoneal macrophages, co-exposure to Pb + LPS induced p42/44 MAPK phosphorylation and TNF-α expression.

In another study on the effect of Pb on TNF-α levels, dairy cows were given Pb orally at 5 mg/kg body weight once daily for four weeks [43]. Blood was collected prior to the initial treatment and again 3, 7, 14, 21 and 28 days later for plasma TNF-α determination. The effect of Pb on TNF-α depended on whether TNF-α was measured in plasma or leukocytes. There were significant differences; plasma TNF-α increased, but in ex vivo leukocytes TNF-α levels did not change when compared with controls. 

## 3. Influence of Pb on Enzymes Involved in Inflammation

### 3.1. Cyclooxygenase 2 (COX-2)

Chang et al. (2011) conducted a study on vascular smooth muscle cells, in which 1 μM Pb induced the production of inflammatory mediators, such as prostaglandin E2, by activating the transcription of genes encoding COX-2 and cytosolic phospholipase A2 with the epidermal growth factor receptor (EGFR), the main receptor responsible for the transduction of Pb-induced signals in these cells [21]. 

The use of an MEK inhibitor PD98059 resulted in the suppression of Pb-induced inflammation, which indicates the involvement of extracellular signal-regulated kinases 1 and 2 p42/44 MAPK in the Pb-induced signal transduction pathway [21]. These findings inspired other researchers to conduct analyses to determine exactly which transcription factors are responsible for the transmission of toxic Pb signals to the nucleus, resulting in COX-2 activation. For example, Chou et al. (2011) researched the mechanism by which EGFR regulates inflammatory genes, such as *COX-2*, using the A431 epidermal carcinoma cell line with a high EGFR expression [20]. The researchers determined the levels of COX-2 mRNA and COX-2 protein after exposing cells to 1 μM Pb(NO_3_)_2_ and found increased activity of the *COX-2* gene and its promoter, and increased expression of the COX-2 protein [20]. Then, in order to identify sites in the *COX-2* gene promoter that are responsible for the effects of the Pb activity, the *COX-2* gene promoter region was examined in the range of −918 to −80 bp [20]. The *COX-2* gene promoter has several potential transcription factor binding sites, such as the cAMP response element (CRE), SP1 (stimulatory protein 1), NF-κB, NFAT/NF-IL6 (nuclear factor of activated t-cells/nuclear factor interleukin 6), and the TATA box [59]. Activation of the gene by these transcription factors usually depends on the cell type and the signaling pathway [20]. 

Lipopolysaccharides can induce expression of the *COX-2* gene by NFAT/NF-IL6 in vascular endothelial cells, while cellular hypoxia activates COX-2, probably via transcription factors such as SP1 and NF-κB [60,61]. Nevertheless, the components required for COX-2 activation by Pb are not yet fully understood. In a study by Chou et al. (2011), removal of the region up to 250 bp (CRE and NFAT/NF-IL6 binding sites remained intact) abolished the response to Pb(NO_3_)_2_ [20]. These results suggest that CRE and NFAT/NF-IL6 are not required to activate the Pb-mediated transcription of the *COX-2* gene. Three other binding sites of other transcription factors (AP2, SP1, and NF-κB) in the *COX-2* gene promoter region (from 918 to 250 bp) were further investigated by Chou et al. (2011) [20]. However, the promoter deletion analysis did not clearly show which binding sites were involved in *the COX-2* gene response to Pb. NFκB inhibitor (BAY 11-7082) was then used, resulting in the Pb-induced inhibition of COX-2 mRNA and protein expression, as well as inhibition of *COX-2* gene promoter activity. Similar results were obtained by blocking NF-κB by siRNA in A431 or CRL1999 cells (smooth muscle cells). These observations confirm the hypothesis that NF-κB is a necessary mediator of the inflammatory cellular response to Pb. EGFR inhibitors (AG1478/PD153035) were also shown to block Pb-induced *COX-2* gene activation and prevented NF-κB translocation into the nucleus, with EGFR siRNA significantly suppressing COX-2 mRNA expression and Pb-induced COX-2 protein synthesis. 

In conclusion, Chou et al. (2011) underlined the role of EGFR/NF-κB in regulating COX-2 activity in cells during exposure to Pb, and showed that studies on EGFR and NF-κB inhibitors may lead to new ways of treating vasculitis caused by occupational and environmental exposure to lead [20]. According to those authors, there may also be other binding sites and transcription factors that can mediate COX-2 expression via alternative pathways. Further research in cell cultures of various organs, as well as in vivo, is needed to determine the molecular mechanisms of COX-2 regulation and pathogenesis of lead toxicity. Stimulation of the A431 epidermal carcinoma cell line by Pb(NO_3_)_2_ (with EGFR receptor) induces translocation of NF-κB transcription factor into the nucleus, affecting *COX-2* gene activity [20].

The effect of Pb on COX-2 was also shown in a study by Simões et al. (2015), in which the thoracic aortas of Sprague Dawley rats were isolated to prepare a primary culture of vascular smooth muscle cells (VSMC) [62]. VSMC, incubated with a 20 μg/dL solution of Pb acetate for 48 h, had an increased level of COX-2 mRNA [62], accompanied by increased expression of COX-2 protein. There were no changes in the expression of COX-1 protein. Earlier observations had shown that changes in COX-2 expression and activity may be affected by MAPK pathways [63,64]. Simões et al. (2015) also checked whether MAPK could be responsible for the observed changes in Pb-induced COX-2 mRNA expression in VSMC. The authors observed a time-dependent stimulation of p42/44 MAPK by Pb (over 30–60 min), but they did not observe this stimulation after long exposures (i.e., after 3 and 24 h). On the other hand, p38 was activated by Pb only after long exposure (i.e., after 24 h). In order to confirm that the p38 and p42/44 MAPK pathways are involved in the activation of COX-2, the authors investigated the effect of inhibitors on COX-2 expression. They showed that U0126 (MEK1 and MEK2 inhibitor) and SB203580 (p38 inhibitor) canceled the induced COX-2 mRNA expression in the VSMC culture. These results may indicate that the activation of proinflammatory COX-2 protein in response to Pb is mediated by signaling pathways p38 and p42/44 MAPK [62]. Nevertheless, the molecular mechanisms and signaling pathways still require further intensive studies. 

Tsai et al. (2015), in a study on human A431 squamous carcinoma cells treated with Pb(NO_3_)_2_, also confirmed the effect of Pb ions on COX-2 expression [65]. Although cells exposed to Pb (1 μM) experienced no changes in the gene expression of DNMT1 and DNMT3b (DNA-methyltransferases), a decrease in the expression of the *DNMT3a* gene was observed. In cells cultured with a methyltransferase inhibitor DAC (5-aza-2′-deoxycytidine), Pb significantly increased the expression of COX-2 mRNA when compared with cells exposed only to Pb. Additionally, the formation of Rb–E2F1 complexes, after binding of Pb to the EGFR receptor, increased the expression of COX-2 by affecting the DMNT3a promoter. Tsai et al. (2015) also indicated inhibited COX-2 transcription in the cell nucleus, resulting in an insufficient amount of enzyme to maintain the repressive methylation of the COX-2 promoter. Consequently, the level of COX-2 mRNA and protein increased. This was confirmed by the results of experiments on EGFR inhibitors (AG1478 and PD153035) and DNMT3a shRNA [65].

### 3.2. Lipoxygenases

The lipoxygenases are lipid-peroxidating enzymes implicated in the cell differentiation and biosynthesis of inflammatory mediators [66]. Human peripheral monocytes do not express a 15LOX, however, in macrophages, the enzyme can be detected in large amounts [67,68]. In macrophages, there are two isoforms of 15LOX, including 15LOX-1, which metabolize arachidonic acid to 15-HETE (15-hydroxy-5,8,11,14 eicosatetraenoic acid) and 12-HETE (12-hydroxy-5,8,10,14 eicosatetraenoic acid) [69,70], and linoleic acid to 9+13-HODE (9-hydroxyoctadecadienoic acid, 13-hydroxyoctadecadienoic acid) [71]. Some authors suggest [72] that end products of 15LOX-1 are only required under homeostatic “resident” conditions, but not during acute inflammation, because subcellular relocalization upon acute inflammation does not occur for 15LOX-1 [72].

F_2_-8α isoprostane, 9-HODE, 13-HODE, and thiobarbituric acid reactive substances (TBARS) are markers of the lipid peroxidation process. Fos-8α isoprostane is formed from arachidonic acid, while 9-HODE and 13-HODE are metabolites of linoleic acid peroxidation. TBARS is considered an indicator of systemic lipid peroxidation.

It is well known that human health may be adversely affected even by low concentrations of heavy metals [73,74], especially in women in the reproductive age [75]. Many studies show that heavy metal contributes to increased oxidative processes [76,77]. 

Pollack et al. (2012) examined the content of Pb and other heavy metals with respect to F_2_-8α isoprostane, 9-HODE, 13-HODE, and TBARS levels in 252 women aged 18–44, in order to assess the relationship between these biomarkers of oxidative stress and hormone levels during a regular menstrual cycle. The concentrations of the analyzed elements in the blood serum of women were relatively low, and resulted from moderate environmental exposure [78]. The results obtained by this team showed that the low blood Pb levels were not associated with an increase in the levels of lipid peroxidation biomarkers (F_2_-8α isoprostane, TBARS, 9-HODE, and 13-HODE) in healthy pre-menopausal women. These results coincide with the outcome of a population study conducted on over 9000 adults above 40 years of age, which showed that exposure to Pb did not correlate with increased levels of inflammatory markers [79].

Kasperczyk et al. (2008) examined populations of healthy and fertile male employees of zinc and lead smelters, occupationally exposed to high levels of heavy metals. Men with blood Pb concentrations below 40 μg/dL had no elevated amounts of lipid peroxidation biomarkers. Decreased sperm motility, most likely because of increased lipid peroxidation, was noticed in men with higher blood lead concentrations (above 40 μg/dL) [80]. 

We found no reports on the changes in the activity of lipoxygenases responsible for the formation of hydroxyoctadecadienoates (HODE) and hydroxyeicosatetraenoates (HETE) in macrophages of peripheral blood in rats exposed to Pb. In our study on two-month-old rats that received 0.1% lead acetate in drinking water during pre- and neonatal periods, we observed (unpublished data) significant increases in blood levels of lipoxygenase (15LOX) products: 15-HETE, 12-HETE, 9-HODE, and 13-HODE. These active products of lipid peroxidation may be responsible for the initiation and propagation of inflammation observed in lead poisoning.

## 4. Effect of Pb on Selected Acute Phase Proteins: C-Reactive Protein (CRP), Haptoglobin, Ceruloplasmin

### 4.1. CRP

CRP is mainly produced in the liver and fat cells in response to proinflammatory factors (IL-1, IL-6). Its major biological function is the ability to recognize microorganisms and damaged host cells, and mediate the process of their elimination by including complementary system and follicular cells [81]. Elevated levels of CRP and a strong positive correlation between blood Pb and CRP levels have been observed in workers exposed to Pb compounds (Pb concentration in whole blood at 29.1 μg/dL) [82].

### 4.2. Haptoglobin

Haptoglobin is responsible for the uptake of free hemoglobin in the blood; under inflammatory conditions, its concentration increases within 48 h to levels many times higher than normal [83]. Kasperczyk et al. (2012) conducted a study in Southern Poland on a group of 192 male employees aged 22–58 who were exposed to Zn and Pb (exposure to Pb lasting from 4 to 37 years), and demonstrated that mean serum haptoglobin increased because of exposure to Pb (haptoglobin concentration in the control group was 117 mg/dL) and that the increase was dependent on the levels of Pb exposure [84]. The haptoglobin concentration increased significantly in the low Pb exposure group (mean Pb-B less than 40 μg/dL, haptoglobin 153 mg/dL), and only slightly in moderate Pb exposure (mean Pb-B 40–50 μg/dL, haptoglobin 132 mg/dL) and high Pb exposure (mean blood Pb > 50 μg/dL, haptoglobin 130 mg/dL) [84].

### 4.3. Ceruloplasmin

Kasperczyk et al. (2012) showed that exposure to Pb increased ceruloplasmin levels. As with haptoglobin, the highest growth was observed in the low Pb exposure group (mean Pb-B Pb < 40 μg/dL, ceruloplasmin 45 mg/dL, compared with 36.6 mg/dL in the control group). The increase in serum ceruloplasmin and haptoglobin may be indicative of the proinflammatory potential of Pb [84].

## 5. Influence of Pb on Immune System Cells

Heavy metals, including Pb, are responsible for the impairment of cells constituting the human immune system.

### 5.1. The Effect of Pb on Lymphocytes T, B, and NK

In vivo research has shown that Pb is immunotoxic and causes depression of humoral immunity [85], increasing host’s susceptibility to bacterial and viral infections [86,87]. In addition, although many studies have also shown that Pb affects the functioning of different types of cells, including immune system cells, the mechanism of this effect is not fully understood. It is known that one of the earliest events in lymphocyte antigen activation is an increase in the activity of protein tyrosine kinase (PTK) [88,89]. An important consequence of this activation is the phospholipase Cγ (PLCγ) tyrosine phosphorylation, which subsequently catalyses the hydrolysis of phosphatidylinositol 4,5-bisphosphate (PIP2) to diacylglycerol and IP3 [90,91]. IP3 results in an increase in intracellular calcium levels [91], which leads to cell transition from G0–G1 to the S-cell phase [92].

In a study by Razani-Boroujerdi et al. (1999), carried out to determine if Pb stimulates lymphocyte proliferation by a similar pathway, the concentration of IP3 was determined in spleen cells after exposure to 50 ppm Pb [93]. Because of Pb interference with the detection of ionized calcium by standard methods (Pb binds to commonly used indicators of ionized calcium), the effect of Pb exposure on PIP2 metabolism was measured by determination of the intracellular IP3 concentration [94].

Within 5 to 7 min, Pb produced a significant increase in IP3 levels in spleen cells, suggesting that Pb can stimulate PLCγ activity [93]. The increase in IP3 concentration was also reported in rat astrocytes exposed to Pb in a study by Dave et al. (1993) [95]. However, Western blot analysis in a study by Razani-Boroujerdi et al. (1999) did not show a significant increase of PTK expression in Pb-treated spleen cells [93]. It is thus unlikely that increased levels of IP3 in spleen cells resulted from PLCγ activation. There are other known IP3 synthesis pathways involving G protein-dependent PLCβ activation [96]. However, there is no direct evidence that Pb induces a G protein-dependent signaling pathway in lymphocytes [93]. The results of that study suggest that the lymphoproliferative activity of Pb may be due to increased synthesis of IP3, and Pb may stimulate PLC activity by a mechanism that is independent of PTK activation, and thus by a mechanism different from the activation of the lymphocyte receptor by antigen [93].

Another study demonstrated that Pb can modify immune reactivity by affecting the direction of T cell precursor lymphocyte differentiation, as naive CD4^+^ T lymphocytes exposed in vitro to Pb had preferential Th2 lymphocyte differentiation [41]. It was also shown that Pb can directly inhibit Th1 cell growth, resulting in decreased levels of IFNγ and IgG_2_a, and that it can also stimulate Th2 lymphocytes, resulting in increased production of interleukin 4 and IgG_4_ and IgE immunoglobulins [41].

A study by Boscolo et al. (1999) showed that Pb has a stimulating effect on CD4^+^ lymphocytes and B lymphocytes, which results in an increase in cytokine production associated with dominant Th2 lymphocyte activation [97]. Another study showed that the number of CD8 lymphocytes involved in the cytotoxic response increases in persons occupationally exposed to Pb (traffic police), accompanied by an increase in IgA levels and a decrease in the number of CD5^+^ B lymphocytes [98].

Boscolo et al. (1997) demonstrated a reduction in the production of T and B lymphocytes (particularly CD4 naive lymphocytes and activated B lymphocytes, types CD3^−^CD25^+^ and CD3^−^HLA^−^DR^+^) [98] and a reduction to 30–50% of NK cells performing the important anti-cancer function, as a result of activities related to the exposure to Pb [99]. An in vitro study on the Pb stimulation of immune cells from a rat spleen showed that concentrations of up to 200 ppm Pb increased thymidine uptake, lymphocyte proliferation, and their maturation and reactivity in allogeneic and syngeneic reactions, and concentrations above 200 ppm inhibited these processes. It follows that Pb has a immunostimulating or immunosuppressive effect, depending on its concentration [93].

Dyatlov and Lawrence (2002), in their aforementioned study, investigated the expression of T cell surface antigens in the blood of Pb-treated BALB/c mice using flow cytometry [30]. The animals were infected with *Listeria monocytogenes* to see how exposure to Pb affected the symptoms of bacterial infection. Compared with the infected mice not exposed to Pb, the Pb-exposed and infected mice showed significant reductions in the expressions of thymic CD4^+^CD8^+^, CD4^+^, CD8^+^, and CD4^−^CD8^−^ [30]. The researchers suggest that this may be related to the presence of a negative zinc balance in the Pb-treated animals in a bacterial infection. Decreased Zn concentrations may inhibit normal T-lymphopoiesis in the thymus [100,101].

Lead causes increased expression of molecules of the major histocompatibility complex class II (MHC II) on the surface of B lymphocytes, thereby influencing their differentiation, possibly by affecting mRNA or post-translational synthesis of cell surface proteins [102].

Many studies show that occupational exposure to Pb has no significant effect on the amount and cytotoxicity of natural killer (NK) cells [103,104], even during concurrent exposure to cadmium [105]. Therefore, we can assume that these lymphocytes are not the main target of lead immunotoxicity. On the other hand, we cannot completely exclude the occurrence of Pb-induced changes within these cells.

### 5.2. Effect of Pb on Macrophages

In macrophages, lead causes dysregulation of proinflammatory cytokine production, such as TNF-α and interleukins 1 and 6, and the preferential production of Th1-type cytokines: IFNγ and IL-2 [106]. Lead can also reduce the synthesis of nitric oxide in macrophages isolated from peripheral blood [106]. 

Macrophages play a key role in both the immune response to lipopolysaccharide (LPS) and parasite infection. As antigen presenting cells (APCs), they appear to be the main target for lead. 

A study by Flohé et al. (2002) [107] investigated the potential influence of lead chloride (PbCl_2_) on the release of cytokines and other inflammatory mediators by bone marrow macrophages (BMMφ) from young female mice. The cells were pre-incubated with PbCl_2_ at various concentrations (0.2–20 μM) and at different times. The macrophages were then stimulated with 10 ng/mL LPS. The supernatant was collected after 7 h to determine tumor necrosis factor α (TNF-α), interleukin 6 (IL-6), and prostaglandin E2 (PGE2), and after 20 h in order to measure the concentrations of interleukins IL-10 and IL-12. Macrophages that were incubated with PbCl_2_ prior to LPS stimulation released more TNF-α, IL-6, IL-12, and PGE2 than control cultures (grown in a pure medium). That effect depended on the contact time and the PbCl_2_ concentration used. The amount of IL-10 determined in the medium of cells previously exposed to PbCl_2_ was lower in relation to the control. These data suggest that increased TNF-α release by macrophages initiated with PbCl_2_ may increase mortality in animals exposed to LPS. Earlier works seem to confirm that Pb increases the susceptibility to bacterial, parasitic, and viral infections in rodents [108] and occupationally exposed workers [109].

Dörpinghaus et al. (2016) investigated how Pb^2+^ influences the cytotoxicity of LPS, focusing on its effect on the synthesis of NO and TNF-α by microglia BV-2 line and macrophages of the RAW264.7 line [110]. These lines are capable of producing both of these factors that contribute to the LPS-induced cytotoxicity. Cells were incubated with various concentrations of lead nitrate Pb(NO_3_)_2_ and lipopolysaccharide (LPS). The amount of TNF-α produced did not change under any of the concentrations of Pb(NO_3_)_2_ used, although a concentration-dependent protective effect of Pb^2+^ against LPS-induced toxicity was found. An increase in the Pb^2+^ concentration in RAW264.7 and BV-2 cells elevated the inhibition of NO release. Pb decreased the LPS-induced expression of the STAT1 transcription factor that is involved in transcription of inducible nitric oxide synthase (iNOS). This results in a reduction in the amount of iNOS enzyme and ultimately leads to a reduced release of NO. Although studies on cell cultures suggest an increase in tolerance to LPS under the influence of Pb^2+^ in vivo, blocking of NO production by Pb^2+^ may cause impairment of pathogen removal, and thus prolong the fight against infections [110]. It is known that LPS induces apoptosis in macrophages [57,111]. Lead can disrupt this action. Concentrations of lead acetate as high as 500 μM turned out to be non-toxic and did not lead to increased apoptosis in peripheral blood mononuclear cells (MNC) [112]. Different results were obtained by Shabani and Rabbani (2000) [113], who tested how different concentrations of nitrate influenced the programmed death in macrophages taken from rat lungs. The authors demonstrated DNA fragmentation even at low Pb concentrations and during short exposure, resulting in macrophage apoptosis. They also noticed an increase in the production of peroxide anion (O_2−_) after only 3 h of exposure. As macrophages are involved in phagocytic and immunoregulatory functions, an increase in superoxide anion production may cause abnormalities in inflammatory reactions and the immune system, as well as lead to tissue damage.

Another mechanism of the influence of Pb on NO levels is presented in Kasten-Jolly and Lawrence (2014) [114]. It suggests that by reducing the amount of intracellular reduced form of glutathione (GSH) and reducing the GSH/GSSG ratio (GSSG being the oxygenated form of glutathione), Pb contributes to increased S-glutathionylation of cysteine residues of the NOS protein. In this way, the production of NO is uncoupling, which in turn results in a decrease in its concentration and increased production of O_2−_. They also indicate that exposure to Pb may affect the polarization of macrophages, which are APC cells, directing it towards the M2 phenotype.

### 5.3. Effect of Pb on Dendritic Cells

Dendritic cells (DCs) are the most important APC for naive lymphocytes. Gao et al. (2007) investigated how lead can modify the development and function of dendritic cells [115]. For this purpose, dendritic cells from the femur and tibia of female BALB/c mice were cultured with the addition of murine granulocyte macrophage-colony stimulating factor (mGM-CSF). The cultures were grown in variants with or without the addition of PbCl_2_ and LPS. Although a smaller proliferation was observed for Pb-treated cells, major histocompatibility class II (MHC-II) had much higher expression than that in cells not exposed to Pb. The authors also point out that Pb-exposed dendritic cells have a reduced expression of inflammatory cytokines and increased expression of IL-10. An increase in the concentration of this cytokine promotes the entry of dendritic cells into the type 2 immune response pathway. The effect of Pb was mainly seen on dendritic cells and not on T lymphocytes, thus the Pb-dependent modification of the dendritic cell function appears to be the main cause of Pb-induced type 2 immunity [115]. 

In 2010, the same team developed a method to examine how toxic substances in the environment can modify the development and function of dendritic cells and, as a consequence, resistance [116]. The researchers chose Pb as a commonly occurring xenobiotic, and dendritic cells taken from PbCl_2_-treated mice were selected as the study group. Next, the function and development of the cells was evaluated. Environmental exposure to Pb caused alteration in the immunophenotype, as well as in the expression of cytokines after cell activation. It was confirmed that the tested cells were able to polarize helper lymphocytes (Th) into Th2, thereby promoting a humoral response.

Pb can also have an effect on Langerhans cells (antigen-presenting cells in the skin) in the production of interleukin 1β and expression of appropriate surface antigens (CD54, CD86, HLA-DR) [117]. It can also modulate the immune response of dendritic cells in the skin [117].

## 6. The Effect of Pb on Immunoglobulins (IgA, IgG, IgM)

Pb can affect not only the cellular, but also the humoral immune response by reduced IgA and IgG production, thereby predisposing individuals to increased inflammatory diseases and cancers [118]. 

Sun et al. (2003) conducted research on the immune system function in pre-school children exposed to environmental concentrations of lead [119]. They examined 38 children with blood Pb ≥ 0.48 μmol/L—the study group—and 35 children with blood lead level ≤0.48 μmol/L—the control group. Analysis of IgG, IgM, and IgE concentrations in sera did not show statistically significant differences between the test group and the control. However, the influence of Pb on the levels of immunoglobulins tested was stronger in women than in men, as IgG and IgM concentrations were significantly lower in the blood serum of the girls in the study group when compared with the control, while an inverse dependence was found for IgE [119]. Similar conclusions were made by Basaran et al. (2000) in their research on a group of 25 men occupationally exposed to Pb [103].

A study by Sarasua et al. (2000) investigated the impact of environmental exposure to Pb on the immune system in over 2000 children and adults [120]. There were no significant differences in the concentrations of immunological markers tested in adults and children over three years of age. However, in children under the age of three, increased levels of serum Pb, mainly those above 15 μg/dL, did correlate with increased levels of IgA, IgG, and IgM.

## 7. The Effect of Pb on Histamine, IgE, and Endothelin

### 7.1. Histamine and Immunoglobin E

In the available literature published after 1990, all authors indicate that Pb may increase the level of IgE in the blood of animals and people.

Farkhondeh et al. (2014) conducted a study in which serum inflammatory mediators and white blood cell (WBC) counts in the blood of sensitized and Pb-exposed guinea pigs were evaluated. Sensitized groups of animals were exposed to 0.1, 0.2, and 0.4 M during and after sensitization of ovoalbumin (OA). The authors showed that inhaled Pb can increase serum total protein, PLA2 (phospholipase A2), IgE, and histamine levels, total and most differential WBC counts in sensitized OA animals, which was more pronounced in animals exposed to Pb [121].

A defining event in asthma is the release of eicosanoids, bioactive lipid metabolites of arachidonic acid (AA). The eicosanoids play an essential role in the inflammatory response, ultimately mediating vasodilation, vascular permeability, broncho-constriction, chemotaxis, and transcription of proinflammatory enzymes. AA, the precursor of eicosanoids, is produced by hydrolysis of membrane phospholipids by PLA2 [122]. Activated inflammatory cells, such as neutrophils and alveolar macrophages, and tracheal epithelial cells, release PLA2 into interstitial or intravascular compartments [122]. PLA2 has been shown to be released from activated mast cells, which are mainly involved in the allergic inflammation of bronchial asthma. Increased PLA2 activity has been demonstrated in serum and bronchoalveolar lavage fluid from asthmatics [121]. 

A study by Heo et al. 1997 on mice exposed to Pb also demonstrated a significant increase in IgE levels [123]. The results of the cited studies suggest that the exposure of asthma patients to environmental Pb contamination could exacerbate the symptoms of the disease [121]. However, the influence of Pb on inflammatory processes is multidirectional, not yet fully understood, and requires further investigation. This seems significant because of increasing environmental pollution with heavy metals. 

The results of a study conducted by Bener et al. (2001) showed a higher incidence of respiratory symptoms and asthma in workers exposed to Pb. In this study, the Pb-exposed group consisted of 100 male industrial workers working in heavy industrial duty, taxi drivers, petrol station gas filling workers, garage workers, chemical products, printing, building, metal industry, or other industrial activities [124]. The non-exposed groups consisted of 100 male workers working in manual jobs not exposed to Pb. Industrial workers had a significantly higher mean Pb-B (77.5 µg/dL) than the non-industrial workers (19.8 µg/dL) [124]. Pb-B exceeded the acceptable level (48 µg/dL) for occupational exposure to Pb, but the Pb-B among non-industrial workers (19.8 µg/dL) was also higher than the threshold level for adults (10 µg/dL) [28]. In this study, the industrial workers had a higher prevalence of respiratory symptoms than non-industrial workers for phlegm, shortness of breath, and diagnosed asthma [124]. Increased respiratory symptoms and decreased pulmonary function tests in workers exposed to Pb were also observed by Khazdair et al. (2012) [125]. 

Additionally, Boskabady et al. (2012) observed that inhaled Pb acetate can increase white blood cell (WBC) count percentages of eosinophil, neutrophil, and basophil in broncho–alveolar lavage, as well as IL-4, despite a reduction in the percentages of lymphocyte IFNγ and in the IFNγ /IL-4 ratio [126]. The same researchers also found that Pb can cause a further increase in total and differential WBC counts of lung lavage, as well as IL-4, with a reduction in the percentage of lymphocyte IFNγ and the IFNγ/IL-4 ratio in methacholine- and ovoalbumin (OA)-sensitized guinea pigs [127]. 

### 7.2. Endothelin

Endothelin-1 (ET-1) is released by the endothelium and exerts a vasodilating effect by the activation of ETA-type receptors in the smooth muscles of blood vessels [128]. Numerous studies have shown that ET-1 is not only a potent vasoconstrictor, but also a proinflammatory agent, for example by inducing COX-2 expression in intraglomerular mesangial cells, in myocardial cells, and in circulating leukocytes [129,130]. In addition, ET-1 stimulates leukocyte adhesion to endothelial cells in the culture and stimulates the release of leukocyte cytokines [131,132,133]. The effect of Pb on the concentration and activity of endothelin is ambiguous. Most authors report an increase in plasma endothelin-3 concentrations in Pb-treated rats, while endothelin-1 remains unchanged [134,135]. Others show an increase in endothelin-1 concentrations in Pb-exposed rats at the same dose (100 ppm) and for the same period (three months) [136].

## 8. Conclusions

The results presented in this review clearly demonstrate that lead (Pb) can play a significant role in the formation and development of inflammation in the body, acting both on the level of gene expression and the synthesis of proinflammatory proteins. It affects, among others, the expression of cytokines (IL8, TNF, IFNγ), the expression and activity of enzymes involved in inflammation (such as COX-2), some acute phase proteins (CRP, haptoglobin, ceruloplasmin), and intracellular molecules mediators (such as histamine and endothelin). It is responsible for the impairment of cells constituting the human immune system (lymphocytes T and B, macrophages, and Langerhans cells). Moreover, Pb can affect not only the cellular, but also the humoral immune response by reducing immunoglobulin production, thereby predisposing individuals to increased inflammatory diseases and cancers. To sum up, the influence of Pb on inflammatory processes is multidirectional, not yet fully understood, and requires further investigation, especially in the situation of growing environmental pollution with heavy metals.

## References

[1] ATSDR (Agency for Toxic Substances and Disease Registry’s) (2007). CERCLA Priority List of Most Hazardous Substances. http://www.atsdr.cdc.gov/cercla.

[2] EP (Environmental Protection) (2005). The Restriction of the Use of Certain Hazardous Substances in Electrical and Electronic Equipment Regulations; No. 2748. http://www.legislation.gov.uk/uksi/2005/2748/pdfs/uksi_20052748_en.pdf.

[3] EP (Environmental Protection) (2009). The Restriction of the Use of Certain Hazardous Substances in Electrical and Electronic Equipment (Amendment) Regulations; No. 581. http://www.legislation.gov.uk/uksi/2009/581/pdfs/uksi_20090581_en.pdf.

[4] EU European Commission Institute for Health and Consumer Protection (2008). Toxicology and Chemical Substances (& ECB): Opinion of the TC NES on the Environment Part of Industry Voluntary Risk Assessments on Lead and Lead Compounds. http://echa.europa.eu/doc/trd_substances/VRAR/Lead/tcnes_opinion/tcnes_opinion_env.pdf.

[5] World Health Organization (WHO) (2009). Global Health Risks: Mortality and Burden of Disease Attributable to Selected Major Risks.

[6] Skoczyńska A., Poręba R., Sieradzki A., Andrzejak R., Sieradzka U. (2002). Wpływ ołowiu i kadmu na funkcje układu immunologicznego. Med. Pr..

[7] Schuster H.P. (1997). Intensywna Terapia w Posocznicy.

[8] Ramella M., Boccafoschi F., Bellofatto K., Md A.F., Fusaro L., Boldorini R., Casella F., Porta C., Settembrini P., Cannas M. (2017). Endothelial MMP-9 drives the inflammatory response in abdominal aortic aneurysm (AAA). Am. J. Transl. Res..

[9] Lee J.C., Laydon J.T., McDonnell P.C., Gallagher T.F., Kumar S., Green D., McNulty D., Blumenthal M.J., Heys J.R., Landvatter S.W. (1994). A protein kinase involved in the regulation of inflammatory cytokine biosynthesis. Nature.

[10] Dietert R.R., Piepenbrink M.S. (2006). Lead and Immune Function. Crit. Rev. Toxicol..

[11] Chibowska K., Baranowska-Bosiacka I., Falkowska A., Gutowska I., Goschorska M., Chlubek D. (2016). Effect of Lead (Pb) on Inflammatory Processes in the Brain. Int. J. Mol. Sci..

[12] Bachmann M.F., Oxenius A. (2007). Interleukin 2: From immunostimulation to immunoregulation and back again. EMBO Rep..

[13] Choi P., Reiser H. (1998). IL-4: Role in disease and regulation of production. Clin. Exp. Immunol..

[14] Iavicoli I., Carelli G., Stanek E.J., Castellino N., Calabrese E.J. (2006). Below background levels of blood lead impact cytokine levels in male and female mice. Toxicol. Appl. Pharmacol..

[15] Baggiolini M., Clark-Lewis I. (1992). Interleukin-8, a chemotactic and inflammatory cytokine. FEBS Lett..

[16] Waugh D.J., Wilson C. (2008). The interleukin-8 pathway in cancer. Clin. Cancer Res..

[17] Lin Y.C., Wei P.L., Tsai Y.T., Wong J.H., Chang C.M., Wang J.Y., Hou M.F., Lee Y.C., Chuang H.Y., Chang W.C. (2015). Pb^2+^ induced IL-8 gene expression by extracellular signal-regulated kinases and the transcription factor, activator protein 1, in human gastric carcinoma cells. Environ. Toxicol..

[18] Lin C.H., Yu M.C., Chiang C.C., Bien M.Y., Chien M.H., Chen B.C. (2013). Thrombin-induced NF-kappaB activation and IL-8/CXCL8 release is mediated by c-Src-dependent Shc, Raf-1, and ERK pathways in lung epithelial cells. Cell. Signal..

[19] Hoffmann E., Dittrich-Breiholz O., Holtmann H., Kracht M. (2002). Multiple control of interleukin-8 gene expression. J. Leukoc. Biol..

[20] Chou Y.H., Woon P.Y., Huang W.C., Shiurba R., Tsai Y.T., Wang Y.S., Hsieh T.J., Chang W.C., Chuang H.Y., Chang W.C. (2011). Divalent lead cations induce cyclooxygenase-2 gene expression by epidermal growth factor receptor/nuclear factor-kappa B signaling in A431carcinoma cells. Toxicol. Lett..

[21] Chang W.C., Chang C.C., Wang Y.S., Weng W.T., Yoshioka T., Juo S.H. (2011). Involvement of the epidermal growth factor receptor in Pb^2+^-induced activation of cPLA_2_/COX-2 genes and PGE_2_ production in vascular smooth muscle cells. Toxicology.

[22] Gillis B.S., Arbieva Z., Gavin I.M. (2012). Analysis of lead toxicity in human cells. BMC Genom..

[23] Zeller I., Knoflach M., Seubert A., Kreutmayer S.B., Stelzmuller M.E., Wallnoefer E., Blunder S., Frotschnig S., Messner B., Willeit J. (2010). Lead contributes to arterial intimal hyperplasia through nuclear factor erythroid 2-related factor-mediated endothelial interleukin 8 synthesis and subsequent invasion of smooth muscle cells. Arterioscler. Thromb. Vasc. Biol..

[24] Kobayashi M., Yamamoto M. (2005). Molecular mechanisms activating the Nrf2-Keap1 pathway of antioxidant gene regulation. Antioxid. Redox Signal..

[25] Roebuck K.A. (1999). Regulation of interleukin-8 gene expression. J. Interferon Cytokine Res..

[26] Yang Y., Zhang X., Fu Y., Yang H. (2014). Leptin and IL-8: Two novel cytokines screened out in childhood lead exposure. Toxicol. Lett..

[27] Roper W.L., Houk V., Falk H., Binder S. (1991). Preventing Lead Poisoning in Young Children: A Statement by the Centers for Disease Control, October 1991.

[28] CDC (2012). Low Level Lead Exposure Harms Children: A Renewed Call for Primary Prevention. https://www.cdc.gov/nceh/lead/acclpp/final_document_030712.pdf.

[29] Turner M.D., Nedjai B., Hurst T., Pennington D.J. (2014). Cytokines and chemokines: At the crossroads of cell signalling and inflammatory disease. Biochim. Biophys. Acta.

[30] Dyatlov V.A., Lawrence D.A. (2002). Neonatal Lead Exposure Potentiates Sickness Behavior Induced by *Listeria monocytogenes* Infection of Mice. Brain Behav. Immun..

[31] Snyder J.E., Filipov N.M., Parsons P.J., Lawrence D.A. (2000). The Efficiency of Maternal Transfer of Lead and Its Influence on Plasma IgE and Splenic Cellularity of Mice. Toxicol. Sci..

[32] Needleman H. (2004). Lead poisoning. Annu. Rev. Med..

[33] Kasten-Jolly J., Heo Y., Lawrence D.A. (2011). Central nervous system cytokine gene expression: Modulation by lead. J. Biochem. Mol. Toxicol..

[34] Klasen H.J., Imfeld K.L., Kirov I.I., Tai L., Gage F.H., Young M.J., Berman M.A. (2003). Expression of cytokines by multipotent neural progenitor cells. Cytokine.

[35] Kishikawa H., Lawrence D.A. (1998). Differential production of interleukin-6 in the brain and spleen of mice treated with lipopolysaccharide in the presence and absence of lead. J. Toxicol. Environ. Health Part A.

[36] Vagaska B., New S.E.P., Alvarez-Gonzalez C., D’Acquisto F., Gomez S.G., Bulstrode N.W., Madrigal A., Ferretti P. (2016). MHC-class-II are expressed in a subpopulation of human neural stem cells in vitro in an IFNγ–independent fashion and during development. Sci. Rep..

[37] Yücesoy B., Turhan A., Ure M., Imir T., Karakaya A. (1997). Effects of occupational lead and cadmium exposure on some immunoregulatory cytokine levels in man. Toxicology.

[38] Heo Y., Parsons P.J., Lawrence D.A. (1996). Lead differentially modifies cytokine production in vitro and in vivo. Toxicol. Appl. Pharmacol..

[39] Kishikawa H., Song R., Lawrence D.A. (1997). Interleukin-12 promotes enhanced resistance to *Listeria monocytogenes* infection of lead-exposed mice. Toxicol. Appl. Pharmacol..

[40] Miller T.E., Golemboski K.A., Ha R.S., Bunn T., Sanders F.S., Dietert R.R. (1998). Developmental exposure to lead causes persistent immunotoxicity in Fischer 344 rats. Toxicol. Sci..

[41] Heo Y., Lee W.T., Lawrence D.A. (1998). Differential effects of lead and cAMP on development and activities of Th1- and Th2-lymphocytes. Toxicol. Sci..

[42] Heo H., Mondal T.K., Gao D., Kasten-Jolly J., Kishikawa H., Lawrence D.A. (2007). Posttranscriptional inhibition of interferon-gamma production by lead. Toxicol. Sci..

[43] Kamińska T., Filar J., Madej E., Szuster-Ciesielska A., Kandefer-Szerszeń M. (1998). Modification of bovine interferon and tumor necrosis factor production by lead in vivo and in vitro. Arch. Immunol. Ther. Exp..

[44] Radbin R., Vahedi F., Chamani J. (2014). The influence of drinking-water pollution with heavy metal on the expression of IL-4 and IFNγ in mice by real-time polymerase chain reaction. Cytotechnology.

[45] Guo T.L., Mudzinski S.P., Lawrence D.A. (1996). The heavy metal lead modulates the expression of both TNF-alpha and TNF-alpha receptors in lipopolysaccharide-activated human peripheral blood mononuclear cells. J. Leukoc. Biol..

[46] Long G.J., Rosen J.F., Schanne F.A. (1994). Lead activation of protein kinase C from rat brain. J. Biol. Chem..

[47] Erickson S.L., de Sauvage F.J., Kikly K., Carver-Moore K., Pitts-Meek S., Gillett N., Sheehan K.C.F., Schreiber R.D., Goeddel D.V., Moore M.W. (1994). Decreased sensitivity to tumor-necrosis factor but normal T-cell development in TNF receptor-2-deficient mice. Nature.

[48] Carpenter A., Evans T.J., Buurman W.A., Bemelmans M.H., Moyes D., Cohen J. (1995). Differences in the shedding of soluble TNF receptors between endotoxin-sensitive and endotoxin-resistant mice in response to lipopolysaccharide or live bacterial challenge. J. Immunol..

[49] Goldfeld A.E., Doyle C., Maniatis T. (1990). Human tumor necrosis factor alpha gene regulation by virus and lipopolysaccharide. Proc. Natl. Acad. Sci. USA.

[50] Cheng Y.J., Yang B.C., Liu M.Y. (2006). Lead increases lipopolysaccharide-induced liver-injury through tumor necrosis factor-α overexpression by monocytes/macrophages: Role of protein kinase C and P42/44 mitogen-activated protein kinase. Environ. Health Perspect..

[51] Dentener M.A., Greve J.W., Maessen J.G., Buurman W.A. (1989). Role of tumour necrosis factor in the enhanced sensitivity of mice to endotoxin after exposure to lead. Immunopharmacol. Immunotoxicol..

[52] Hofsli E., Bakke O., Nonstad U., Espevik T. (1989). A flow cytometnc and immunofluorescence microscopic study of tumor necrosis factor production and localization in human monocytes. Cell. Immunol..

[53] Hewett J.A., Jean P.A., Kunkel S.L., Roth R.A. (1993). Relationship between tumor necrosis factor-alpha and neutrophils in endotoxin-induced liver injury. Am. J. Physiol..

[54] Leist M., Gantner F., Künstle G., Bohlinger I., Tiegs G., Bluethmann H., Wendel A. (1995). The 55-kD tumor necrosis factor receptor and CD95 independently signal murine hepatocyte apoptosis and subsequent liver failure. Mol. Med..

[55] Schuchmann M., Varfolomeev E.E., Hermann F., Rueckert F., Strand D., Koehler H., Strand S., Lohse A.W., Wallach D., Galle P.R. (2003). Dominant negative MORT1/FADD rescues mice from CD95 and TNF-induced liver failure. Hepatology.

[56] Künstle G., Leist M., Uhlig S., Revesz L., Feifel R., MacKenzie A., Wendel A. (1997). ICE-protease inhibitors block murine liver injury and apoptosis caused by CD95 or by TNF-alpha. Immunol. Lett..

[57] Comalada M., Xaus J., Valledor A.F., Lopez-Lopez C., Pennington D.J., Celada A. (2003). PKC epsilon is involved in JNK activation that mediates LPS-induced TNF-alpha, which induces apoptosis in macrophages. Am. J. Physiol. Cell-Physiol..

[58] Beutler B., Cerami A. (1989). The biology of cachectin/TNF—A primary mediator of the host response. Annu. Rev..

[59] Appleby S.B., Ristimaki A., Neilson K., Narko K., Hla T. (1994). Structure of the human cyclo-oxygenase-2 gene. Biochem. J..

[60] Inoue H., Yokoyama C., Hara S., Tone Y., Tanabe T. (1995). Transcriptional regulation of human prostaglandin-endoperoxide synthase-2 gene by lipopolysaccharide and phorbol ester in vascular endothelial cells. Involvement of both nuclear factor for interleukin-6 expression site and cAMP response element. J. Biol. Chem..

[61] Xu Q., Ji Y.S., Schmedtje J.F. (2000). Sp1 increases expression of cyclooxygenase-2 in hypoxic vascular endothelium, Implications for the mechanisms of aortic aneurysm and heart failure. J. Biol. Chem..

[62] Simões M.R., Aguado A., Fiorim J., Silveira E.A., Azevedo B.F., Toscano C.M., Zhenyukh O., Briones A.M., Alonso M.J., Vassallo D.V. (2015). MAPK pathway activation by chronic lead-exposure increases vascular reactivity through oxidative stress/cyclooxygenase-2-dependent pathways. Toxicol. Appl. Pharmacol..

[63] Ohnaka K., Numaguchi K., Yamakawa T., Inagami T. (2000). Induction of cyclooxygenase-2 by angiotensin II in cultured rat vascular smooth muscle cells. Hypertension.

[64] Wang H., Xi S., Xu Y., Wang F., Zheng Y., Li B., Li X., Zheng Q., Sun G. (2013). Sodium arsenite induces cyclooxygenase-2 expression in human uroepithelial cells through MAPK pathway activation and reactive oxygen species induction. Toxicol. In Vitro.

[65] Tsai Y.T., Chang C.M., Wang J.Y., Hou M.F., Wang J.M., Shiurba R., Chang W.C., Chang W.C. (2015). Function of DNA Methyltransferase 3a in Lead (Pb^2+^)-Induced *Cyclooxygenase-2* Gene. Environ. Toxicol..

[66] Kuhn H., Walther M., Kuban R.J. (2002). Mammalian arachidonate 15-lipoxygenases structure, function, and biological implications. Prostaglandins Other Lipid Mediat..

[67] Chaitidis P., O’Donnell V., Kuban R.J., Bermudez-Fajardo A., Ungethuem U., Kuhn H. (2005). Gene expression alterations of human peripheral blood monocytes induced by medium-term treatment with the TH2-cytokines interleukin-4 and -13. Cytokine.

[68] Conrad D.J., Kuhn H., Mulkins M., Highland E., Sigal E. (1992). Specific inflammatory cytokines regulate the expression of human monocyte 15-lipoxygenase. Proc. Natl. Acad. Sci. USA.

[69] Dobrian A.D., Lieb D.C., Cole B.K., Taylor-Fishwick D.A., Chakrabarti S.K., Nadler J.L. (2011). Functional and pathological roles of the 12- and 15-lipoxygenases. Prog. Lipid Res..

[70] Wittwer J., Hersberger M. (2007). The two faces of the 15-lipoxygenase in atherosclerosis. Prostaglandins Leukot. Essent. Fatty Acids.

[71] Shureiqi I., Lippman S.M. (2001). Lipoxygenase modulation to reverse carcinogenesis. Cancer Res..

[72] Poeckel D., Zemski Berry K.A., Murphy R.C., Funk C.D. (2009). Dual 12/15- and 5-lipoxygenase deficiency in macrophages alters arachidonic acid metabolism and attenuates peritonitis and atherosclerosis in ApoE knock-out mice. J. Biol. Chem..

[73] Bazan N.G., Colangelo V., Lukiw W.J. (2002). Prostaglandins and other lipid mediators in Alzheimer’s disease. Prostaglandins Other Lipid Mediat..

[74] Oberg B.P., McMenamin E., Lucas F.L., McMonagle E., Morrow J., Ikizler T.A., Himmelfarb J. (2004). Increased prevalence of oxidant stress and inflammation in patients with moderate to severe chronic kidney disease. Kidney Int..

[75] Vahter M., Berglund M., Akesson A., Lidén C. (2002). Metals and women’s health. Environ. Res..

[76] Stohs S.J., Bagchi D. (1995). Oxidative mechanisms in the toxicity of metal ions. Free Radic. Biol. Med..

[77] Ercal N., Gurer-Orhan H., Aykin-Burns N. (2001). Toxic metals and oxidative stress part I: Mechanisms involved in metal-induced oxidative damage. Curr. Top. Med. Chem..

[78] Pollack A.Z., Schisterman E.F., Goldman L.R., Mumford S.L., Perkins N.J., Bloom M.S., Rudra C.B., Browne R.W., Wactawski-Wende J. (2012). Relation of Blood Cadmium, Lead, and Mercury Levels to Biomarkers of Lipid Peroxidation in Premenopausal Women. Am. J. Epidemiol..

[79] Songdej N., Winters P.C., McCabe M.J., van Wijngaarden E. (2010). A population-based assessment of blood lead levels in relation to inflammation. Environ. Res..

[80] Kasperczyk A., Kasperczyk S., Horak S., Ostałowska A., Grucka-Mamczar E., Romuk E., Olejek A., Birkner E. (2008). Assessment of semen function and lipid peroxidation among lead exposed men. Toxicol. Appl. Pharmacol..

[81] Boncler M., Luzak B., Watała C. (2006). Znaczenie białka C-reaktywnego w patofizjologii miażdżycy. Role of C-reactive protein in atherogenesis. Postepy Hig. Med. Dosw..

[82] Khan D.A., Qayyum S., Saleem S., Khan F.A. (2008). Lead-induced oxidative stress adversely affects health of the occupational workers. Toxicol. Ind. Health.

[83] Szczeklik A. (2005). Choroby Wewnętrzne. Tom II.

[84] Kasperczyk A., Prokopowicz A., Dobrakowski M., Pawlas N., Kasperczyk S. (2012). The Effect of Occupational Lead Exposure on Blood Levels of Zinc, Iron, Copper, Selenium and Related Proteins. Biol. Trace Elem. Res..

[85] Luster M.I., Faith R.E., Kimmel C.A. (1978). Depression of humoral immunity in rats following chronic developmental lead exposure. J. Environ. Pathol. Toxicol..

[86] Hemphil F.E., Kaeberle M.L., Buck W.B. (1971). Lead suppression of mouse resistance to Salmonella typhimurium. Science.

[87] Gainer J.H. (1974). Lead Aggravates Viral Disease and Represses the Antiviral Activity of Interferon Inducers. Environ. Health Perspect..

[88] Chan A., Desai D., Weiss A. (1994). The role of tyrosine kinases and protein tyrosine phosphatases in the antigen receptor signal transduction. Annu. Rev. Immunol..

[89] Cambier J.C., Pleiman C.M., Clark M.R. (1994). Signal transduction by the B cell antigen receptor and its co-receptors. Annu. Rev. Immunol..

[90] Robey E., Allison J.P. (1995). T cell activation: Integration of signals from the antigen receptor and costimulatory molecules. Immunol. Today.

[91] Berridge M.J. (1995). Inositol trisphosphate and calcium signaling. Ann. N. Y. Acad. Sci..

[92] Crabtree G.R., Clipstone N.A. (1994). Signal transmission between the plasma membrane and nucleus of T-lymphocytes. Annu. Rev. Biochem..

[93] Razani-Boroujerdi S., Edwards B., Sopori M.L. (1999). Lead stimulate lymphocyte proliferation through enhanced T cell-B cell interaction. J. Pharmacol. Exp. Ther..

[94] Schanne F.A., Moskal J.R., Gupta P.K. (1989). Effect of lead on intracellular free calcium ion concentration in a presynaptic neuronal model: 19F-NMR study of NG108-15 cells. Brain Res..

[95] Dave V., Vitarella D., Aschner J.L., Fletcher P., Kimelberg H.K., Aschner M. (1993). Lead increases inositol 1,4,5-trisphospate levels but does not interfere with calcium transient in primary rat astrocytes. Brain Res..

[96] Gilman A.G. (1987). G-protein: Transducer of receptor-generated signals. Annu. Rev. Biochem..

[97] Boscolo P., Di Gioacchino M., Sabbioni E., Benvenuti F., Conti P., Reale M., Bavazzano P., Giuliano G. (1999). Expression of lymphocyte subpopulations, cytokine serum levels, and blood and urinary trace elements in asymptomatic atopic men exposed to an urban environment. Int. Arch. Occup. Environ. Health.

[98] Boscolo P., Di Gioacchino M., Spanò A., Di Giacomo F., Ballone E., D’Isidoro G., Cavallucci E., Giuliano G. (1997). Trace elements in biological samples and immunologic parameters in environmentally exposed populations (preliminary study). Giornale Italiano di Medicina del Lavoro ed Ergonomia.

[99] Boscolo P., Di Gioacchino M., Bavazzano P., White M., Sabbioni E. (1997). Effects of chromium on lymphocyte subsets and immunoglobulins from normal population and exposed workers. Life Sci..

[100] Victery W., Miller C.R., Zhu S.Y., Goyer R.A. (1987). Effect of different levels and periods of lead exposure on tissue levels and excretion of lead, zinc, and calcium in the rat. Fundam. Appl. Toxicol..

[101] McCabe M.J., Lawrence D.A. (1990). The heavy metal lead exhibits b cell-stimulatory factor by enhancing B cell Ia expression and differentiation. J. Immunol..

[102] McCabe M.J., Lawrence D.A. (1991). Lead, a major environmental pollutant, is immunomodulatory by its differential effects on CD4+ T cell subsets. Toxicol. Appl. Pharmacol..

[103] Basaran N., Undeger U. (2000). Effects of lead on immune parameters in occupationally exposed workers. Am. J. Ind. Med..

[104] Mishra K.P., Singh V.K., Rani R., Yadav V.S., Chandran V., Srivastava S.P., Seth P.K. (2003). Effect of lead exposure on the immune response of some occupationally exposed individuals. Toxicology.

[105] Yücesoy B., Turhan A., Ure M., Imir T., Karakaya A. (1997). Simultaneous effects of lead and cadmium on NK cell activity and some phenotypic parameters. Immunopharmacol. Immunotoxicol..

[106] Krocova Z., Macela A., Kroca M., Hernychova L. (2000). The immunomodulatory effect(s) of lead and cadmium on the cells of immune system in vitro. Toxicol. In Vitro.

[107] Flohé S.B., Brüggemann J., Herder C., Goebel C., Kolb H. (2002). Enhanced proinflammatory response to endotoxin after priming of macrophages with lead ions. J. Leukoc. Biol..

[108] Lawrence D.A. (1981). In vivo and in vitro effects of lead on humoral and cell-mediated immunity. Infect. Immun..

[109] Horiguchi S., Endo G., Kiyota I., Teramoto K., Shinagawa K., Wakitani F., Tanaka H., Konishi Y., Kiyota A., Ota A. (1992). Frequency of cold infections in workers at a lead refinery. Osaka City Med. J..

[110] Dörpinghaus M., Brieger A., Panichkina O., Rink L., Haase H. (2016). Lead ions abrogate lipopolysaccharide-induced nitric monoxide toxicity by reducing the expression of STAT1 and iNOS. J. Trace Elem. Med. Biol..

[111] Xaus J., Comalada M., Valledor A.F., Lloberas J., López-Soriano F., Argilés J.M., Bogdan C., Celada A. (2000). LPS induces apoptosis in macrophages mostly through the autocrine production of TNF-α. Blood.

[112] De la Fuente H., Portales-Pérez D., Baranda L., Díaz-Barriga F., Saavedra-Alanís V., Layseca E., González-Amaro R. (2002). Effect of arsenic, cadmium and lead on the induction of apoptosis of normal human mononuclear cells. Clin. Exp. Immunol..

[113] Shabani A., Rabbani A. (2000). Lead nitrate induced apoptosis in alveolar macrophages from rat lung. Toxicology.

[114] Kasten-Jolly J., Lawrence D.A. (2014). Lead modulation of macrophages causes multiorgan detrimental health effects. J. Biochem. Mol. Toxicol..

[115] Gao D., Mondal T.K., Lawrence D.A. (2007). Lead effects on development and function of bone marrow-derived dendritic cells promote Th2 immune responses. Toxicol. Appl. Pharmacol..

[116] Gao D., Lawrence D.A. (2010). Dendritic cells in immunotoxicity testing. Methods Mol. Biol..

[117] Aiba S., Terunuma A., Manome H., Tagami H. (1997). Dendritic cells differently respond to haptens and irritants by their production of cytokines and expression of co-stimulatory molecules. Eur. J. Immunol..

[118] Anetor J.I., Adeniyi F.A. (1998). Decreased immune status in Nigerian workers occupationally exposed to lead. Afr. J. Med. Med. Sci..

[119] Sun L., Hu J., Zhao Z., Li L., Cheng H. (2003). Influence of exposure to environmental lead on serum immunoglobulin in preschool children. Environ. Res..

[120] Sarasua S.M., Vogt R.F., Henderson L.O., Jones P.A., Lybarger J.A. (2000). Serum immunoglobulins and lymphocyte subset distributions in children and adults living in communities assessed for lead and cadmium exposure. J. Toxicol. Environ. Health Part A.

[121] Farkhondeh T., Boskabady M.H., Kohi M.K., Sadeghi-Hashjin G., Moin M. (2014). Lead exposure affects inflammatory mediators, total and differential white blood cells in sensitized guinea pigs during and after sensitization. Drug Chem. Toxicol..

[122] Rothenberg M., Saito H., Peebles R.S. (2017). Advances in mechanisms of allergic disease in 2016. J. Allergy Clin. Immunol..

[123] Heo Y., Lee W.T., Lawrence D.A. (1997). In vivo the environmental pollutants lead and mercury induce oligoclonal T cell responses skewed toward type-2 reactivities. Cell. Immunol..

[124] Bener A., Almehdi A.M., Alwashc R. (2001). A pilot survey of blood lead levels in various types of workers in the United Arab Emirates. Environ. Int..

[125] Khazdair M.R., Boskabady M.H., Afshari R., Dadpour B., Behforouz A., Javidi M., Abbasnezhad A., Moradi V., Tabatabaie S.S. (2012). Respiratory symptoms and pulmonary function tests in lead exposed workers. Iran. Red. Crescent Med. J..

[126] Boskabady M.H., Karimi G.R., Samarghandian S., Farkhondeh T. (2012). Tracheal responsiveness to methacholine and ovalbumin; and lung inflammation in guinea pigs exposed to inhaled lead after sensitization. Ecotoxicol. Environ. Saf..

[127] Farkhondeh T., Boskabady M.H., Koohi M.K., Sadeghi-Hashjin G., Moin M. (2013). The effect of lead exposure on selected blood inflammatory biomarkers in guinea pigs. Cardiovasc. Hematol. Disord. Drug Targets.

[128] Russell F.D., Skepper J.N., Davenport A.P. (1997). Davenport Detection of endothelin receptors in human coronary artery vascular smooth muscle cells but not endothelial cells by using electron microscope autoradiography. J. Cardiovasc. Pharmacol..

[129] Sugimoto T., Haneda M., Sawano H., Isshiki K., Maeda S., Koya D., Inoki K., Yasuda H., Kashiwagi A., Kikkawa R. (2001). Endothelin-1 induces cyclooxygenase-2 expression via nuclear factor of activated T-cell transcription factor in glomerular mesangial cells. J. Am. Soc. Nephrol..

[130] Molero L., Farré J., García-Mendez A., Jiménez Mateos-Caceres P., Carrasco M.C., Millás I., Navarro F., Córdoba M., Casado S., López-Farré A. (2003). Endothelin-1 induced proinflammatory markers in the myocardium and leukocytes of guinea-pigs: Role of glycoprotein IIB/IIIA receptors. Cardiovasc. Res..

[131] López Farré A., Riesco A., Espinosa G., Digiuni E., Cernadas M.R., Alvarez V., Montón M., Rivas F., Gallego M.J., Egido J. (1993). Effect of endothelin-1 on neutrophil adhesion to endothelial cells and perfused heart. Circulation.

[132] McCarron R.M., Wang L., Stanimirovic D.B., Spatz M. (1993). Endothelin induction of adhesion molecule expression on human brain microvascular endothelial cells. Neurosci. Lett..

[133] McMillen M.A., Huribal M., Cunningham M.E., Kumar R., Sumpio B.E. (1995). Endothelin-1 increases intracellular calcium in human monocytes and causes production of interleukin-6. Crit. Care Med..

[134] Khalil-Manesh F., Gonick H.C., Weiler E.W., Prins B., Weber M.A., Purdy M.E. (1993). Lead-induced hypertension: Possible role of endothelial factors. Am. J. Hypertens..

[135] Gonick H.C., Ding Y., Bondy S.C., Ni Z., Vaziri N.D. (1997). Lead-induced hypertension: Interplay of nitric oxide and reactive oxygen species. Hypertension.

[136] Khalil-Manesh F., Gonick H.C., Weiler E.W.J., Prins B., Weber M.A., Purdy R., Ren Q. (1994). Effect of chelation treatment with dimercaptosuccinic acid (DMSA) on lead-related blood pressure changes. Environ. Res..

